# Wide-angle non-uniform optical phased array using compact and efficient antenna design

**DOI:** 10.1038/s41598-024-54016-w

**Published:** 2024-02-15

**Authors:** Omar E. Elsheikh, Mohamed A. Swillam

**Affiliations:** Nanophotonics Research Laboratory, Department of Physics, The American University in Cairo, New Cairo Avenue, 11835 Cairo Governorate, Egypt

**Keywords:** Integrated optics, Nanophotonics and plasmonics, Integrated optics, Nanophotonics and plasmonics

## Abstract

In the need for a more compact and efficient optical phased array with a wide steering beam for LIDAR applications, a wide steering array with high resolution is desirable. However, in the published work, a trade-off is often made for one over another. Apodized grating antennas have shown good efficiency with a compact size and wide beam profile, which improve optical phased array beam steering capability and are also compatible with the CMOS silicon photonics process. A promising studies shows enhancement in steering range with good resolution utilizing a non-uniform optical phased array. In this work, we present two highly efficient optical antennas with 94% and 93.5% upward power at the center frequency for the first and second antenna respectively, exceeding state-of-the-artwork to the best of our knowledge, and wide full-width half maximum of 8.88° x 78.05° and 7.53° x 69.85° in elevation and azimuthal planes, respectively. Both antennas provide a broad bandwidth across the 1400–1700 nm wavelength range with more than 80% efficiency in the S, C, and L bands. To overcome the limited scan ranges and small aperture size, a two-dimensional non-uniform array of 10 × 10 elements is utilized to increase the beam steering capability. A genetic algorithm is used to optimize the position of array elements, resulting in an aliasing-free array with a wide steering range of 160° with beam width 0.5° and consistent −11 dB maximum side lobe level across the steering range.

## Introduction

The phased array, first proposed in the early 1800s, is a valuable tool for communications systems due to its features, including shaped antenna patterns, dynamic range, beam control, high gain, directivity, in-band linearity, adaptive interference cancellation, and angle-measurement accuracy^1^. Microwaves and optical waves are electromagnetic waves with comparable propagation properties. But optical waves are far shorter than microwaves. Optical phased arrays (OPAs) are inspired by microwave phased arrays, which guide light beams without hardware movement which could replace Current commercial beam steering systems that use mechanical scanning, making them perfect for important technology like light detection and ranging (LIDAR) also makes them difficult to integrate into small platforms and meet the speed requirements of such technology. LiDAR has various uses, including autonomous driving, terrestrial mapping, aerospace, and optical sensing^2^. This article discusses the design and simulation of optical antennas and OPA that could be useful for LiDAR systems, particularly automotive ones.

Si photonics (SiP) uses semiconductor industry infrastructure and expertise to allow low-cost, high-performance, and scalable optical applications. It has enabled high-speed optical transceiver modules for data centers and long-haul networks, achieving commercial success. LIDAR technologies are largely SiP-based. Si photonics for LiDAR replaces a complex free-space optical system with numerous separate optical components with a chip-scale silicon platform solution. A complex optical system for LiDAR is apparently a perfect target to tackle, as it involves many challenges such as beam quality, beam steering range, power consumption, reliability, and cost.

Mechanical scanning, MEMS scanning, flash imaging, and OPA are all LiDAR mapping methods, however OPA may be best for SiP. After the initial SiP demonstration in OPAs in 2009^3^, numerous researchers have developed compact and efficient OPAs with small antenna elements. With enough antennae, an OPA may guide a beam in 2D without a lens system and construct any beam patterns. The number of phase shifters and antennas and optical performance are generally limited by design complexity, power consumption, and cost. Most researchers want excellent resolution and steering range in OPAs for practical LIDAR applications. However, current designs generally sacrifice one for the other^4,5^.

Waveguiding material, beam divergence, beam steering range, 2D beam steering, antenna element performance, and antenna array design must be considered while designing a SiP OPA. Waveguiding material impacts optical loss, bandwidth, polarisation dependency, and device compatibility. SiP LiDAR systems typically employ C-band (1530-1565 nm) or O-band (1260–1360 nm) to utilize foundries’ process design kit, supply chain, and product development experience^5^. Beam steering range is the highest beam deflection angle. It sets the LiDAR FOV. A FOV > 120^o^ is ideal for automobile applications. Aliasing, diffraction envelope, and phase shifter tuning range limit beam steering. 2D beam steering allows horizontal and vertical steering. This can be done with a 2D OPA or two cascaded 1D OPAs with orthogonal polarisation. A 2D OPA is more complicated but has less insertion loss than two 1Ds.

The efficiency of an antenna’s grating design has been demonstrated in previous studies^6–10^, particularly in terms of compact size and efficiency for directing output beam. Given its significance as a crucial component in LIDAR systems, this study aims to enhance the design proposed by Khajavi et al.^11^ Their design, which builds upon the work of Melati et al, achieved an upward efficiency of 89% while preserving a compact form factor. Despite the great efficiency and compact size of their design, it encounters certain challenges that we have endeavored to address and successfully overcome. The authors employed unfamiliar substances possessing refractive indices of 2.64 and 2.39 in order to enhance the upward efficiency of the antenna, while yet preserving its compact dimensions. Insufficient details were provided regarding the 2D OPA employed in their study.To address these concerns, we enhanced the design by incorporating diverse partial etching along the grating and implementing a bottom Bragg-Reflector. This modification resulted in an improved diffraction efficiency of the antenna, increasing it from the original maximum efficiency of 89–94%. Furthermore, this enhancement allowed for a size reduction, resulting in dimensions of 6.5$\mu$m x 3$\mu$m.

In addition, a silicon nitride (SiN) overlay was employed in conjunction with silicon, since it exhibits greater compatibility with a wide range of complementary metal-oxide-semiconductor (CMOS) processes, also SiN deposition and etching methods are well-developed and can produce high-quality films with various thicknesses, refractive indices, and optical losses. Consequently, a second antenna was produced, boasting a diffraction efficiency of 93.5% with dimensions measuring 7.5$\mu$m $\times$ 3$\mu$m. While the integration of SiN in this particular design may encounter fabrication challenges, previous studies^12^ have shown that SiN deposition can be achieved using different techniques, including low-pressure chemical vapour deposition (LPCVD), plasma-enhanced chemical vapour deposition (PECVD), and atomic layer deposition (ALD). Each technique possesses distinct advantages and disadvantages in terms of film quality, stress control, conformality, and scalability. In addition, SiN etching can be performed using either wet or dry techniques. Wet etching typically exhibits isotropic behavior and demonstrates a notable preference for silicon dioxide, however, it can induce undercutting and surface roughness. Dry etching typically exhibits anisotropic characteristics, enabling the attainment of elevated aspect ratios and the creation of smooth sidewalls. However, it is important to note that this method may also result in detrimental effects such as damage and contamination. The use of silicon nitride (SiN) waveguides featuring partially-etched lateral trenches has demonstrated potential in mitigating the detrimental effects of bending loss in waveguides characterized by tiny radii of curvature. The concept was demonstrated by Kastenmeier et al.^13^ through the utilization of plasma chemical dry etching (CDE) to remove SiN from a silicon substrate. The study shows that the utilization of partially-etched trenches can enhance the effective refractive index contrast and effectively limit a greater amount of light within the core region.

The simulation results for the two proposed antennas were acquired using a commercially available finite difference time domain (FDTD) method-based software program developed by Lumerical Inc., a company based in Canada. Subsequently, we aim to investigate the configuration of a two-dimensional optical phased array (OPA) utilizing the suggested antenna structures. To attain a high resolution and wide steering range, researchers have proposed a promising method known as the non-uniform optical phased array (OPA)^14,15^. This approach involves optimizing the sparse pitches among the antenna elements, resulting in an increase in the effective optical aperture and a reduction in grating lobes or aliasing effects. Additionally, efforts are made to minimize the side lobe level. In our study, we utilized a genetic algorithm (GA) to optimize a 2D array consisting of 10 x 10 elements. The objective was to suppress grating lobes and enhance the steering capability of the array. As a result, we were able to achieve a 160^0^ alias-free steering range. The design specifics of the two suggested antennas are presented in Section “Design setup”, followed by the simulation results in Section “Antenna simulation results”. Section “2D array simulation and optimization” of the paper delves into the simulation of a two-dimensional (2D) array and explores how the application of the GA optimization technique might enhance the array’s steering range and resolution. Section “Genetic algorithm optimization” serves as the concluding section, providing a comprehensive summary of the work.

## Design setup

**Figure 1:**
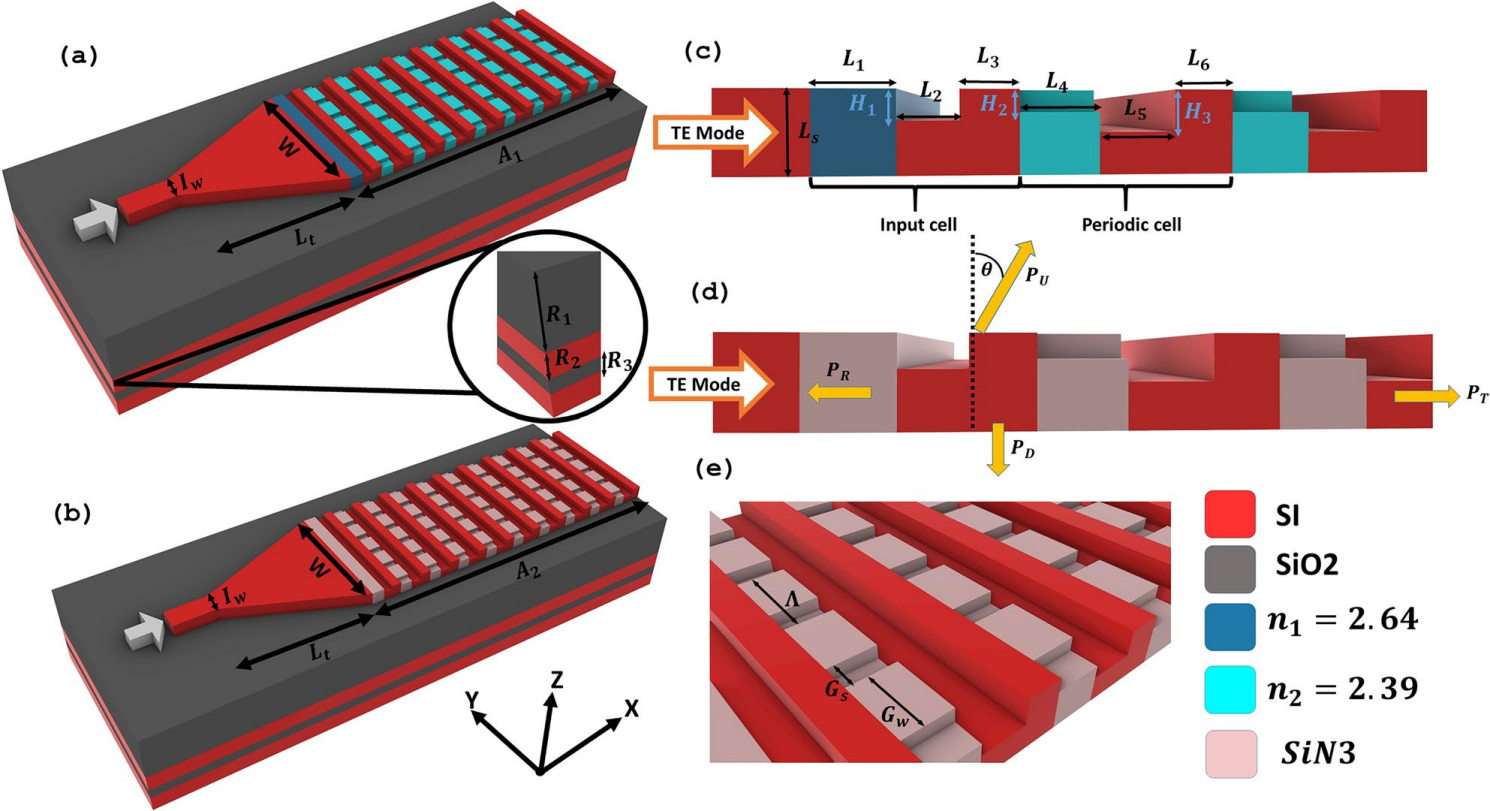
3D unit cells of (**a**) first antenna with grating made of materials of refractive indices $n_1$ = 2.64 and $n_2$ = 2.39, (**b**) second antenna with grating made of Si and SiN. Both antennas have a down Bragg reflector with thicknesses $R_1$, $R_2$ and $R_3$. Side view of (**c**) first antenna, (**d**) second antenna. Both structures are excited with input TE mode, optimized grating lengths ($L_1$, $L_2$, $L_3$, $L_4$, $L_5$, $L_6$), thicknesses ($H_1$, $H_2$, $H_3$) and emitted power-up ($P_U$), reflected power ($P_R$), power transmitted ($P_T$) and power down ($P_D$) . (**e**) Grating parameters: width ($G_w$), space ($G_s$) and period ($\Lambda$).

Figure 1 shows the two unit cells of the proposed structures and their details. The first antenna unit cell (Fig. 1a) size is 6.5$\mu$m x 3$\mu$m while the second antenna (Fig. 1b) has a size of 7.5$\mu$m x 3$\mu$m, with input waveguide of thickness $L_s$ = 300 nm and excited with TE mode for both antennas. The initial step involves employing identical length ratios as those of the original antenna. Subsequently, we proceed with the optimization of grating lengths and etches through a brute-force approach. The significance of the input cell in regulating the upward radiation or emission of power has been noticed. As a result, in both designs, the intentional omission of partial etching was implemented in the input cell to optimize the radiation of power in the upward direction. Additionally, in the cell following the input waveguide in both antennas, we conducted experimental adjustments to the lengths and etches to facilitate efficient diffraction of the remaining power upwards. The deliberate decisions made by the individuals involved ultimately result in the values that are reported in Table 1.

**Figure 2:**
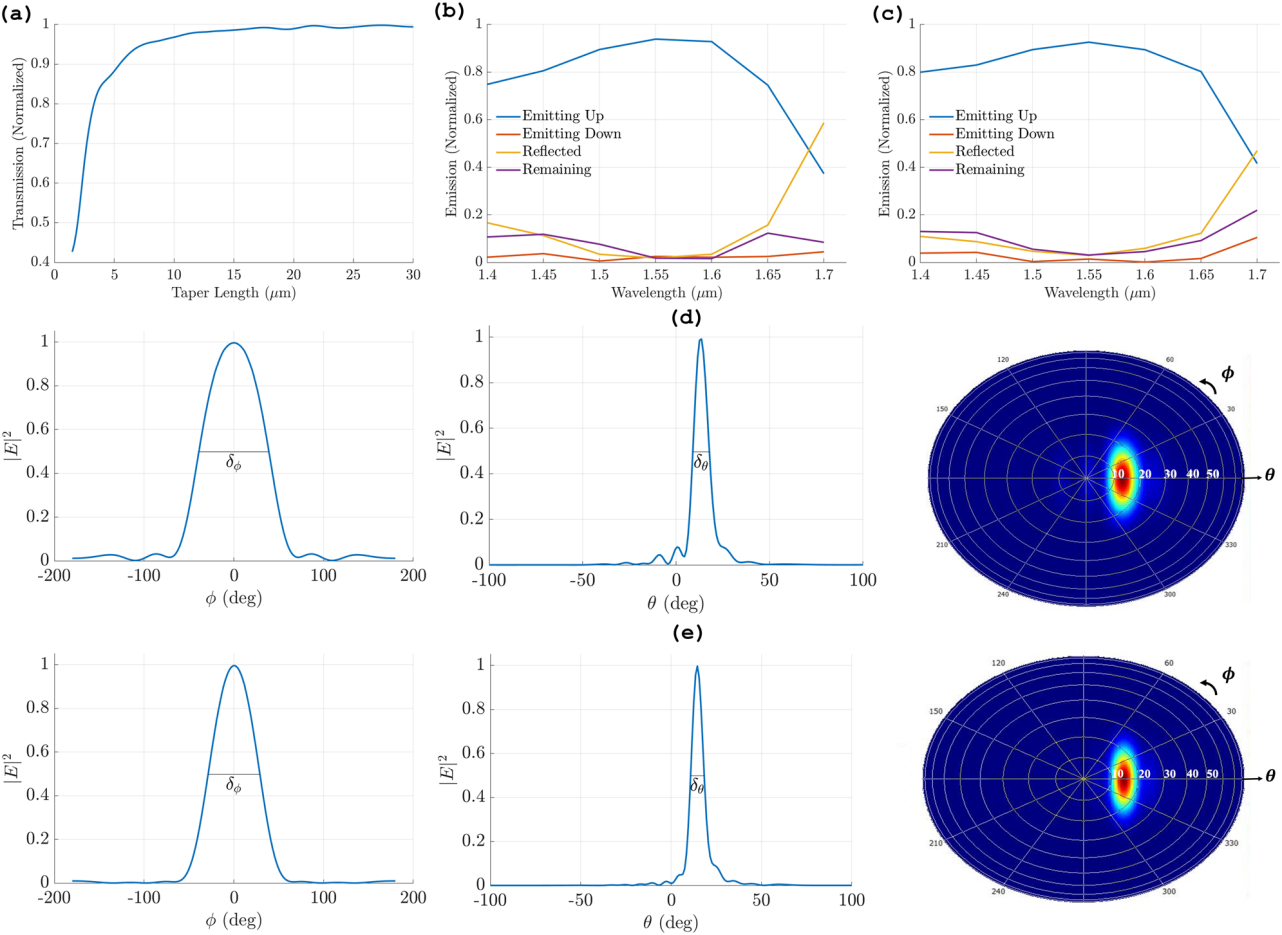
(**a**) Normalized transmission of the adiabatic tapered waveguide as a function of the taper length. The normalized optical radiation efficiency of (**b**) first antenna, (**c**) second antenna. FWHM ($\delta$) at azimuthal angle ($\phi$)= 0^0^ and far field radiation of (**d**) first antenna $\delta _\phi$= 78.05^0^ and $\delta _\theta$= 8.88^0^ at $\theta$= 13^0^, (**e**) second antenna $\delta _\phi$= 57.75^0^ and $\delta _\theta$= 7.53^0^ at $\theta$= 14.5^0^.

An adiabatic tapered waveguide is used to provide a smooth transition of light from an input fiber to a single-mode output waveguide without radiating outside the waveguide or converting to other higher-order waveguide modes^16^. The width of the input waveguide is 500 nm. The length of the linear taper is optimized until the maximum output power is stable. As shown in Fig. 2a, a 15$\mu$m taper can efficiently couple the TE mode from the input fiber to the antenna with an efficiency 98%. The full unit cell of the first antenna with the optimized taper results in only 21.5$\mu$m x 3$\mu$m while for the second antenna, the full unit cell results in 22.5$\mu$m x 3$\mu$m.

The two structures start with a one-step input cell and a periodic cell. The first antenna (Fig. 1c) composed of two parts: the first one with refractive index $n_1$ = 2.64 with $L_1$ = 280 nm, while the second part has partial etching of thickness $H_1$ = 110 nm, which helps in breaking the up-down symmetry of the grating antenna and assists in increasing the diffraction efficiency utilizing the constructive-destructive interference^17^, and $L_2$ = 225 nm, and $L_3$ = 210 nm. The periodic part has lengths $L_4$ = 270 nm with refractive index $n_2$ = 2.39 and partial etching $H_2$ = 80 nm, $L_5$ = 276 nm with etching $H_3$ = 150 nm, and $L_6$ = 220 nm. The second antenna was constructed with the same lengths and etching thickness but with SiN material overlays, as shown in Fig. 1d. As for the grating width ($G_w$) and grating space ($G_s$) are kept as the original design by values of 164 nm and 236 nm, respectively, along with a grating period ($\Lambda$) of 400 nm, are employed depicted in Fig. 1e.

The two antennas are situated on a silicon-on-insulator (SOI) platform, which consists of a 1.042$\mu$m SiO_2_ substrate and a 1$\mu$m silica cladding. Partial etching was found to improve the upper efficiency of the initial design by roughly 2%, resulting in an efficiency of around 91%. However, the dimensions of the design remained the same at 7.6$\mu$m x 4.5$\mu$m. Therefore, there was a desire to further optimize the dimensions and efficiency of the design. One notable improvement that has been explored in the literature involves the incorporation of a bottom mirror. Zhang et al.^18^ conducted a study wherein Distributed Bragg Reflectors (DBR) were employed at the bottom of the grating antenna. This addition resulted in a significant enhancement of the unidirectional transmission efficiency, increasing it from 46% to 95%. Similarly, Khajavi et al.^7^ achieved a noteworthy emission efficiency of 82% by employing near-field phase engineering, and this accomplishment was facilitated through the incorporation of a lower Bragg reflector within the framework of the antenna configuration. Nevertheless, it is important to acknowledge that the inclusion of the Bragg reflector in the production process introduces a heightened level of complexity to the design fabrication. In order to optimize upward efficiency, In the proposed design, a silicon-based brag reflector is employed in conjunction with two silicon mirrors which further pushes the efficiency by another 3%. Each mirror has a thickness of $\lambda _{Si}/4$ and is separated by a layer of SiO_2_ with a thickness of $\lambda _{SiO_2}/4$^19^ as represented in the inset of Fig. 1a.

The aforementioned arrangement results in a notable increase in efficiency, exceeding 94%, specifically at a wavelength of 1550 nm. Furthermore, this feature facilitates the achievement of a more compact design, leading to dimensions measuring 6.5$\mu$m x 3$\mu$m. This signifies a decrease in both the length and width by 16% and 40% respectively. Additionally, the incorporation of SiN in place of the impractical materials utilized in the original design allows for a 93.5% upper efficiency at a wavelength of 1550 nm, with dimensions of 7.5$\mu$m × 3$\mu$m, when combined with the bottom mirror.

**Table 1:** Optimized design parameters used for the two antennas.

Parameter	Value ($\mu$m)	Parameter	Value ($\mu$m)	Parameter	Value ($\mu$m)
$L_1$	0.28	$H_1$	0.110	$R_2$	$\lambda _{Si}$/4
$L_2$	0.225	$H_2$	0.80	$R_3$	$\lambda _{SiO_2}$/4
$L_3$	0.210	$H_3$	0.150	$I_w$	0.5
$L_4$	0.270	$G_s$	0.164	*W*	3
$L_5$	0.276	$G_w$	0.236	$L_t$	15
$L_6$	0.220	$\Lambda$	0.4	$A_1$	6.5
$L_s$	0.3	$R_1$	1.042	$A_2$	7.5

## Antenna simulation results

Investigating the response of the two designs in the S, C, and L bands by varying the source wavelength from 1400 nm to 1700 nm and measuring the output emissions. Figure 2b shows the first antenna response with a maximum upward efficiency 94% at 1550 nm, while for the second antenna (Fig. 2c) maximum upward efficiency is 93.5% at 1550 nm. As shown, the efficiency of both antennas exceeds 80% from 1460 nm to 1630 nm which demonstrates the wide working band capability of the two proposed structures.

Figure 2d shows simulation results for the characteristics of the first antenna at a wavelength of 1,550 nm, the antenna maximum diffraction is at an angle 13^o^ in elevation ($\theta ^o$) plane with a wide full width half maximum (FWHM) $\delta _\phi$= 78.05^o^ x $\delta _\theta$= 8.88^o^ in the azimuth and elevation planes, respectively. For SiN overlay antenna response at 1,550 nm, Fig. 2(e) shows a FWHM of $\delta _\phi$= 69.85^o^ x $\delta _\theta$= 7.53^o^ in azimuth and elevation planes, with maximum diffraction at 14.5^o^ at $\theta$ in the far field. To compare the achieved results, Table 2 presents a comparison with the state-of-the-art reported optical antennas in terms of size and diffraction efficiency. The results indicate that the proposed antennas exhibit the highest diffraction efficiency, to the best of our knowledge, in the smallest compact size.

**Table 2:** Comparison between proposed antennas and state-of-the-art optical antennas.

Work	Element size ($\mu$m)	Upper emission at 1550 nm (%)
This work (1st antenna)	6.5 × 3	94
This work (2nd antenna)	7.5 × 3	93.5
^6^ (2023)	500 × 1	92
^14^ (2023)	4.2 × 2	41.4
^7^ (2022)	3.1 × 1.75	82
^20^ (2021)	150 × 2	73
^8^ (2021)	7.09 × 3	91
^11^ (2021)	7.6 × 4.5	89
^21^ (2021)	146 × 2.25	45
^22^ (2021)	18 × 2.25	40

## 2D array simulation and optimization

**Figure 3:**
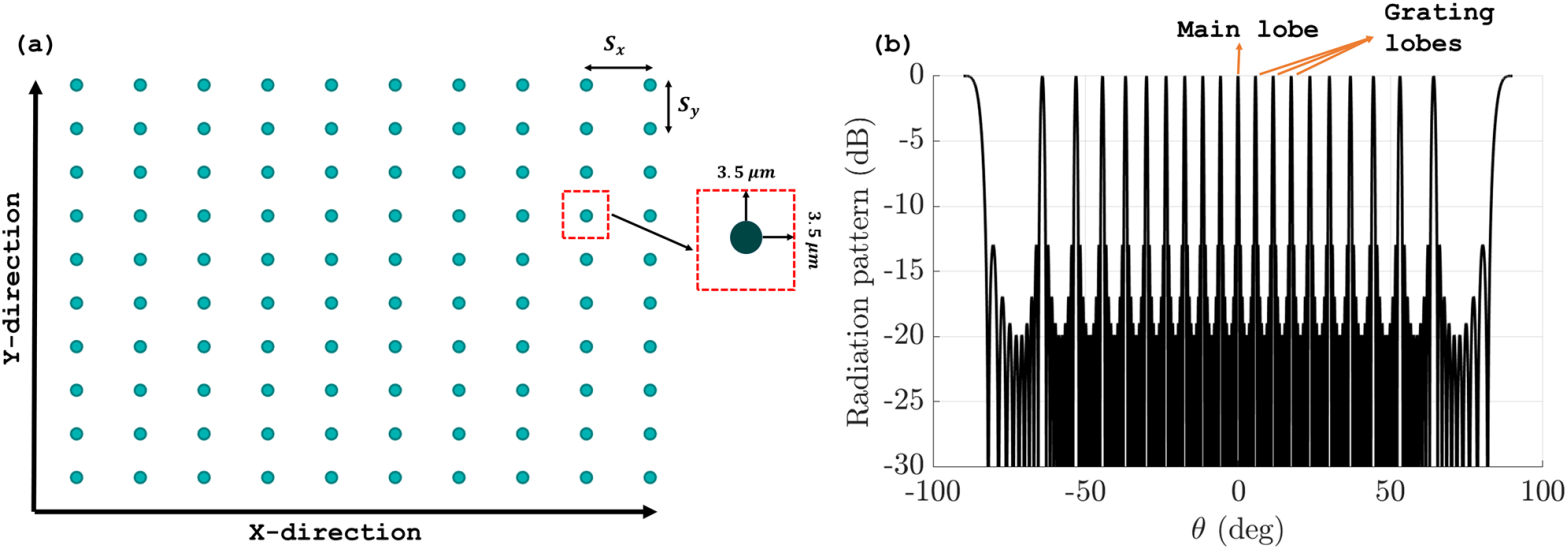
(**a**) Non-optimized uniform rectangular array grid of 10x10 elements with element spacing ($S_x$ = $S_y$ = 10$\mu$m) and each element allowed to move for 3.5$\mu$m in x and y directions. (**b**) Non-optimized radiation pattern for antenna array (log scale).

The efficient design of 2D OPA, especially in LIDAR applications, necessitates the use of compact elements to achieve an efficient chip size, maximize the number of elements, and enhance the overall steering range of the chip. A 2D array of the first antenna, as it is the most compact and efficient, is simulated with 10 x 10 elements in a uniform rectangular array (URA). An element spacing of 10$\mu$m is used since it is hard to use the conventional $\lambda$/2 spacing without overlapping of antenna pattern and also to let each antenna element move freely during the optimization without getting too close to the neighboring one. Figure 3a show the initial grid of the array with element spacing $S_x$ = $S_y$ = 10$\mu$m in x and y directions respectively. The choice of 10$\mu$m as the spacing between the antenna elements is not the optimal one but rather a test case to demonstrate the effectiveness of our proposed algorithm. Even with such a large spacing, which increases the aliasing phenomenon and creates unwanted lobes in the radiation pattern, our algorithm can successfully suppress these lobes with high efficiency. The spacing can be reduced further to form a more compact array with the aid of the proposed compact antennas. The far-field radiation pattern of this initial array is shown (in log scale) in Fig. 3b. As shown, many grating lobes appeared in the visible region due to the large spacing used; this constraints the steering ability of the array, limiting the practicality of the OPA. To get rid of these grating lobes, the position of each element in the array is perturbed within a 3.5$\mu$m by 3.5$\mu$m area in the x and y directions, as shown in Fig. 3a (red dotted square). However, the solution space of this problem is huge; therefore, a GA is employed as it has the capability to effectively explore a vast solution space^23^.

### Genetic algorithm optimization

**Figure 4:**
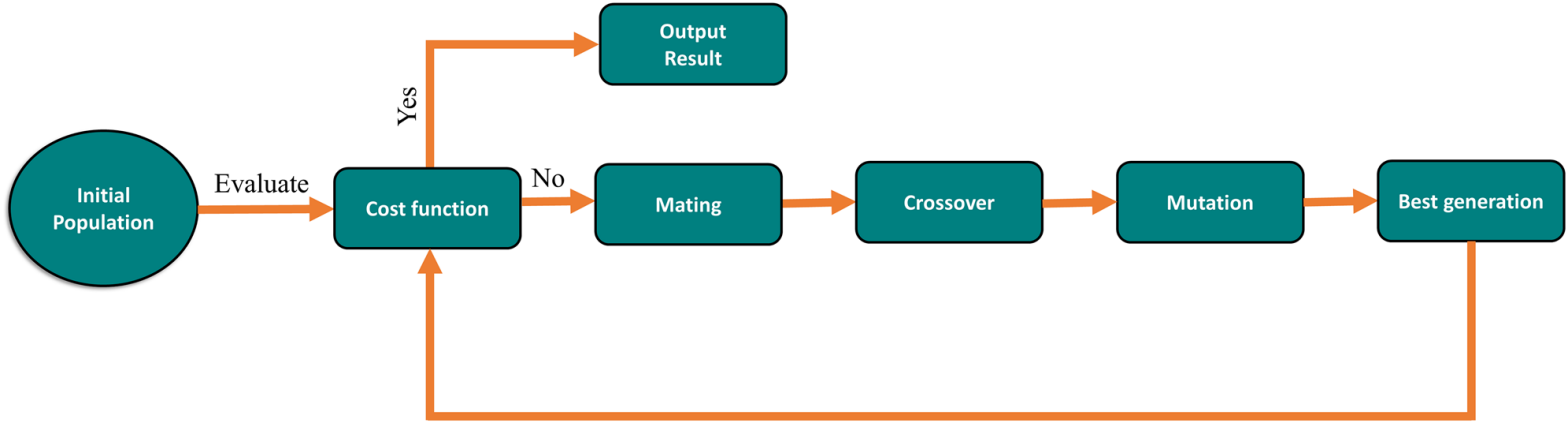
Genetic algorithm flowchart used to optimize the antenna array.

GA is a powerful tool for optimization and search problems that mimic the process of natural selection and serves as an alternative method to ordinary local search algorithms. GA has been used in the field of electromagnetics, especially in antenna design and optimization^24^ and antenna array problems^14,23,25^. Inspired by the biological mechanisms of inheritance and adaptation, GA is a different way of solving optimization problems than conventional local search methods. In biology, genetics deals with how traits are passed on and vary among living organisms. These traits can be modified by processes such as selective breeding and genetic engineering. Evolution is closely related to genetics, as it causes changes in the genetic makeup of a population through natural selection, genetic drift, mutation, and migration. These processes enable a population to become more fit for its environment, or in other words, to optimize its performance. A similar idea can be applied to numerical optimization, where the goal is to find a good solution within some constraints on the variables. A chromosome is an input to an objective function, which represents our goal from the optimization, and its output is called the cost when minimizing. A chromosome is made up of genes or individual variables that have certain alleles or values. Figure 4 shows a flowchart of the used algorithm. The algorithm is initialized with a random population that is represented with a matrix where each row corresponds to a chromosome, and then each chromosome is fed to the cost function to be evaluated. The cost function is the most important part of the algorithm, as it is called many times when evaluating the outlay of population members. The fitness of the cost function is calculated based on the value of the array factor for the 2D array given in Eq. (1)

1 $$\begin{aligned} {} \begin{aligned} \hspace{5 cm} f = \quad \min _{P} \quad&\max _{\theta \in \Omega } |AF(\theta , \phi )| \\ \text {s.t.} \quad&\Omega = \theta :-90 \le \theta \le -\delta \theta \quad \text {or} \quad \delta \theta \le \theta \le 90 \end{aligned} \end{aligned}$$

where beamwidth (BW) = 2 $\delta \theta$ = FWHM of the main lobe and AF($\theta$, $\phi$) is the array factor of the uniform rectangular array and given in Eq. (2)^26^

2 $$\begin{aligned} {} \hspace{3 cm} AF(\theta , \phi ) = \sum _{a=1}^{N_x} e^{j \;k \;S_x(\sin {\theta }\sin {\phi } - \sin {\theta _s\sin {\phi _s}})} * \sum _{b=1}^{N_y} e^{j \;k \;S_y \;(\sin {\theta }\sin {\phi } - \sin {\theta _s\sin {\phi _s}})} \end{aligned}$$

where $\phi$ is the azimuthal angle, $\theta$ is the elevation angle, *k* is the wave number, which equals $2\pi$/$\lambda$, $S_x$ and $S_y$ are the element spacing in the x and y directions, respectively. After calculating the AF for each generation, ranking is done to determine which population produced the maximum side lobe level below our termination criteria. If the evaluated population doesn’t meet the criteria for termination, the set of chromosomes goes through mating, which allows the fittest member of the population to be selected, then a crossover is done between the survived chromosomes to generate offspring, a mutation is done to maintain diversity within the population and prevent premature convergence, and at the end, a remeasure of the maximum side lobe is done until reaching the termination condition or reaching the end of the generation count.

The GA is applied here to randomly redistribute the position of each antenna element in a rectangular phased array, either vertically or horizontally within a constrained box of 3.5$\mu$m by 3.5$\mu$m (Fig. 3a), to break the symmetry and eliminate the grating lobes. We are aware that these constraints may cause cross-talk between antenna elements, but each antenna has little radiation and high directivity, making it insignificant. Our optimization method takes into account the array elements’ non-uniformity to reduce cross-talk. Non-uniformity is required to get the optimum performance while minimizing cross-talk. The algorithm represents the phased array as a list of element position perturbations, which is the chromosome to be optimized. All other parameters of the array, such as the number of elements, uniform spacing, and steering angle, are kept constant until the algorithm reaches the termination condition or the number of generations.

**Figure 5:**
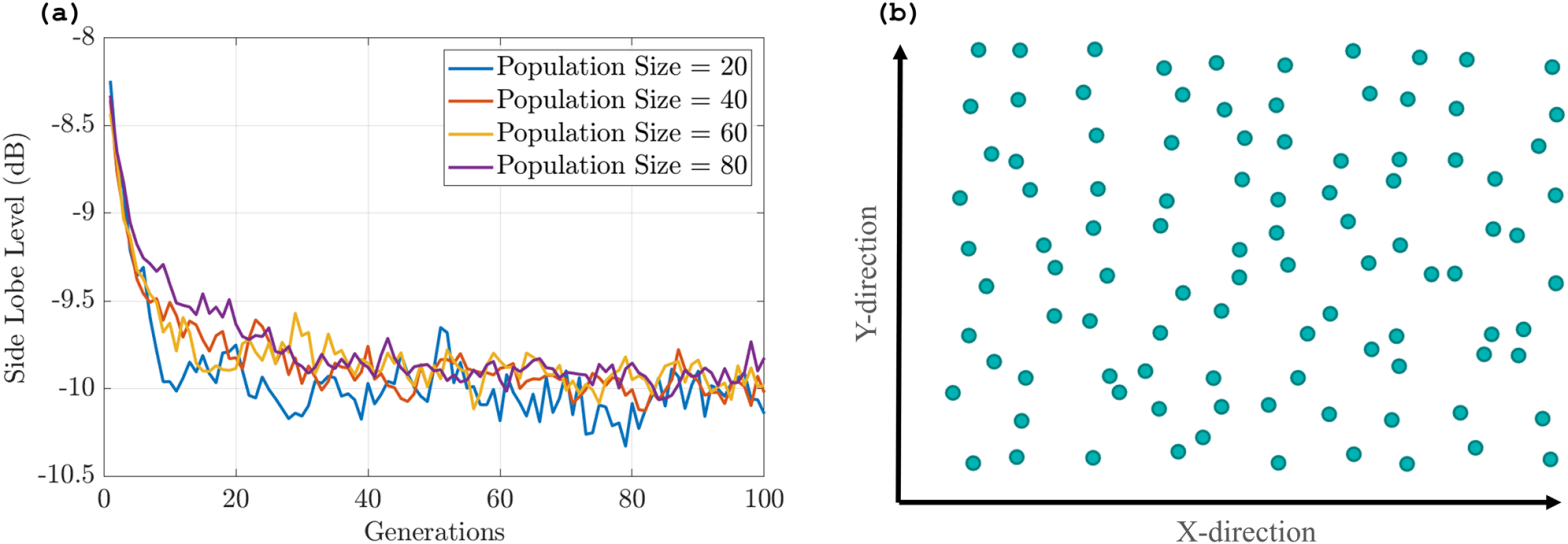
(**a**) Maximum side lobe level for population size: 20 (Blue), 40 (Red), 60 (Yellow) and 80 (Violet). (**b**) Optimized positions resulted from generating algorithm.

**Figure 6:**
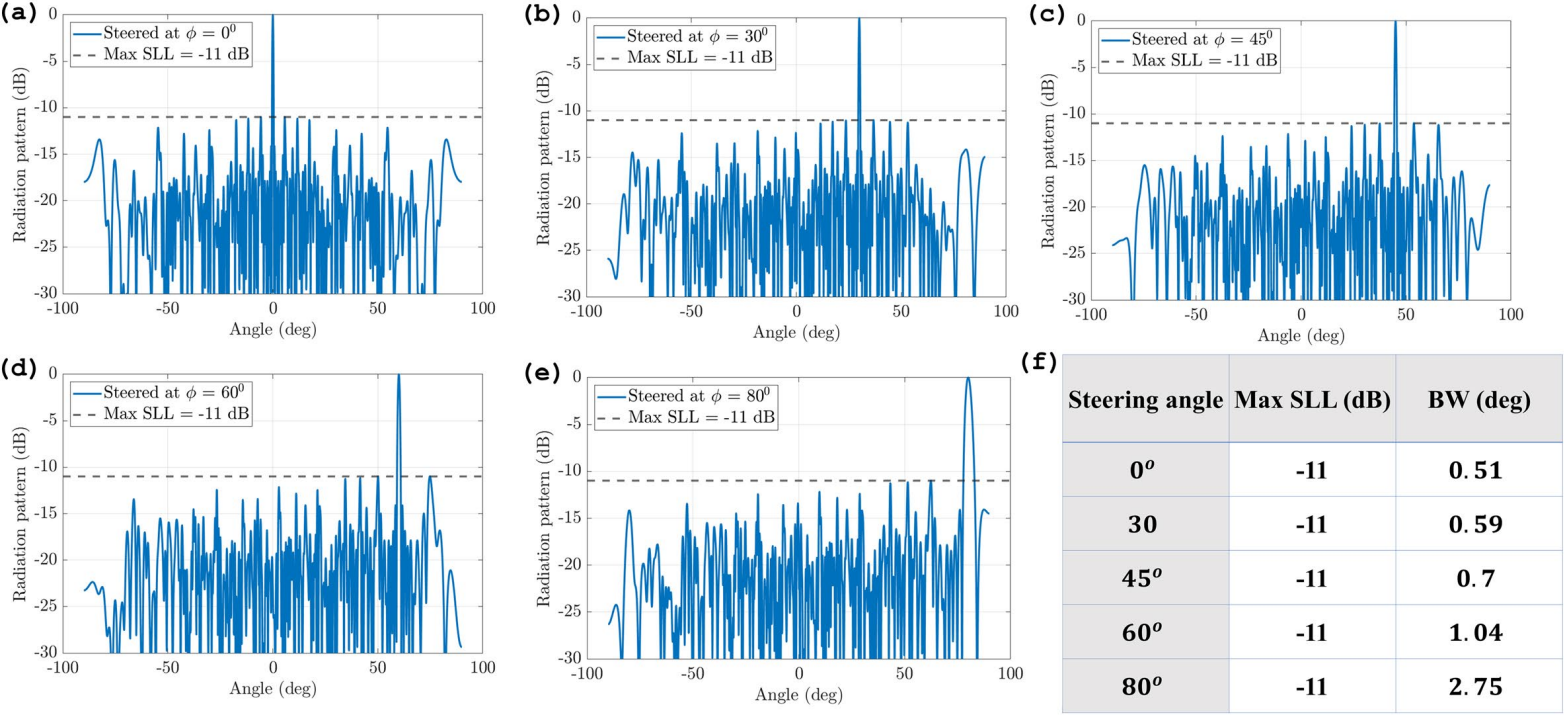
Radiation pattern of optimized array (log scale) at $\theta$ = (**a**) 0^0^, (**b**) 30^o^, (**c**) 45^0^, (**d**) 60^o^ and (**e**) 80^o^. (**f**) Resulted max side lobe level (SLL) and beamwidth (BW) of the main lobe of the array for each steered angle.

To use the GA effectively, we need to decide on several parameters, such as the population size, crossover rate, mutation rate, and termination condition^26^. A binary GA is used with a cost function for measuring the maximum side lobe level (SLL) for different positions of the antenna elements in the array, which is formulated and fed into the algorithm with a termination condition for this phased array set to −11 dB. Otherwise, the algorithm is run for 100 generations, and the crossover and mutation rates are set to 0.99 and 0.006, respectively. We ran the optimization for different population sizes and observed that the performance was similar across all of them (see Fig. 5a). Therefore, we chose a population size of 20 across all the simulations in order to minimize the simulation time. The optimization of the array results in a non-uniform array (Fig. 5b) with optimized positions in the x and y directions (see Table S1 in Supplementary data). These optimized array produce a far-field radiation pattern (Fig. 6a) with suppressed grating lobes comparable to the initial array with a maximum SLL of −11 dB, which is a significant improvement over the conventional uniform array. The beamwidth of the main lobe is also narrowed to 0.51^o^, which indicates a higher angular resolution and better target detection capability. These performance metrics can be further improved by increasing the number of antennas in the array.

In order to test the maximum steering angle of the array, the optimized array is steered to different angles of $\theta$ equal: 0^o^, 30^o^, 45^o^, 60^o^ and 80^o^ and the array pattern at each angle is shown in Fig. 6b–e. The elevation pattern cuts illustrate that even when steering the main beam, no grating lobes are observed in the visible region, even at angles as large as $\theta$ = 80^0^. Figure 6f shows that beamwidth gradually increases as the angles of $\theta$ become larger; however, this effect can be mitigated by increasing the number of elements. Based on these results, it appears that the phenomenon of aliasing has minimal impact on the steering range. However, when the steering angle exceeds 80 degrees, the main lobe of the array begins to exhibit increased bandwidth and an altered shape, thereby resulting in a steering range of ± 80 degrees or 160^0^. A comparison of the performance of an optimized non-uniform 2D array with previously reported work in terms of steering range is summarized in Table 3.

**Table 3:** Comparison between proposed optical phased array design and state-of-the-art work in terms of steering range.

Work	Steering range
This work	160°
^14^ (2023)	53°
^27^ (2023)	100°
^28^ (2022)	53.7°
^29^ (2022)	91.8°
^9^ (2021)	140°
^30^ (2021)	77.4°
^18^ (2019)	40°

## Conclusion

This study presents two designs for compact dielectric antennas, measuring 6.5$\mu$m x 3$\mu$m and 7.6$\mu$m x 3$\mu$m, respectively. These designs exhibit upward diffraction efficiency of 94% and 93.5%, and full-width half maximum of 8.88^0^ x 78.05^0^ and 7.53^0^ x 69.85^0^, respectively. Notably, these performance metrics exceed those reported for antennas of similar dimensions range. Table 2 compares the proposed antennas to the current state-of-the-art antennas, including their specifications. The comparison shows that the proposed antennas exhibit high efficiency with compact sizes compared to other reported antennas. A non-uniform 2D array is studied and optimized with the genetic algorithm optimization technique, which results in an alias-free wide steering array with a suppressed side lobe level to a maximum of -11 dB. The 2D array is capable of exhibiting a wide steering range of 160^o^ within the visible region. In conclusion, we propose a compact design of an optical antenna that can be easily fabricated using standard Silicon photonics technology and can be conveniently manufactured and employed to enhance the performance of OPAs and LIDAR technologies across diverse applications.

### Supplementary Information

Supplementary Table S1.

## Data Availability

The datasets used and/or analyzed during the current study are available from the corresponding author upon reasonable request.
